# Modulating the Electronic Structure and Basal-Plane Adsorption of Janus CrSSe through Mechanochemical Activation

**DOI:** 10.1021/acsomega.6c04409

**Published:** 2026-10-07

**Authors:** Juan Gomez Quispe, Pedro Alves da Silva Autreto

**Affiliations:** Center of Natural and Human Sciences, 425753 Federal University of ABC, Santo Andre 09210-580, Sao Paulo, Brazil

## Abstract

Mechanochemical activation offers a reversible and chemically noninvasive route to tune the basal-plane reactivity of two-dimensional catalysts. Here, spin-polarized density functional theory calculations are employed to investigate strain- and curvature-engineered Janus CrSSe for the hydrogen evolution reaction, comparing H* adsorption on the CrSSe-S and CrSSe-Se surfaces and extending the analysis to zigzag CrSSe-NT­(*n*,0) nanotubes. HER-relevant H adsorption thermodynamics are evaluated within the computational hydrogen electrode framework using the adsorption free energy, Δ*G*_ads_, and the H* binding energy, *E*_b_. Pristine 2D CrSSe exhibits strongly non-thermo-neutral H* adsorption with a pronounced polarity dependence. In-plane strain markedly reconstructs the electronic structure, with biaxial tension inducing metallicity and spin polarization and driving Δ*G*_ads_ close to thermo-neutral values. Curvature introduces an additional polarity-dependent degree of freedom: S-terminated nanotubes exhibit spin-polarized metallic behavior, whereas Se-terminated nanotubes remain semiconducting and converge to zero-net-spin states. A pronounced structural–magnetic crossover between CrSSe-NT-S­(20,0) and (21,0) is accompanied by an abrupt change in H* binding, revealing a direct coupling between curvature, magnetism, and chemisorption. Moderate axial tensile strain of 3–5% further tunes this curvature-dependent adsorption response and brings Δ*G*_ads_ closer to the thermo-neutral regime over a broad range of nanotube diameters. Overall, these results establish strain, curvature, and Janus polarity as coupled physical descriptors for controlling the electronic, magnetic, and hydrogen adsorption properties of CrSSe nanostructures.

## Graphical abstract

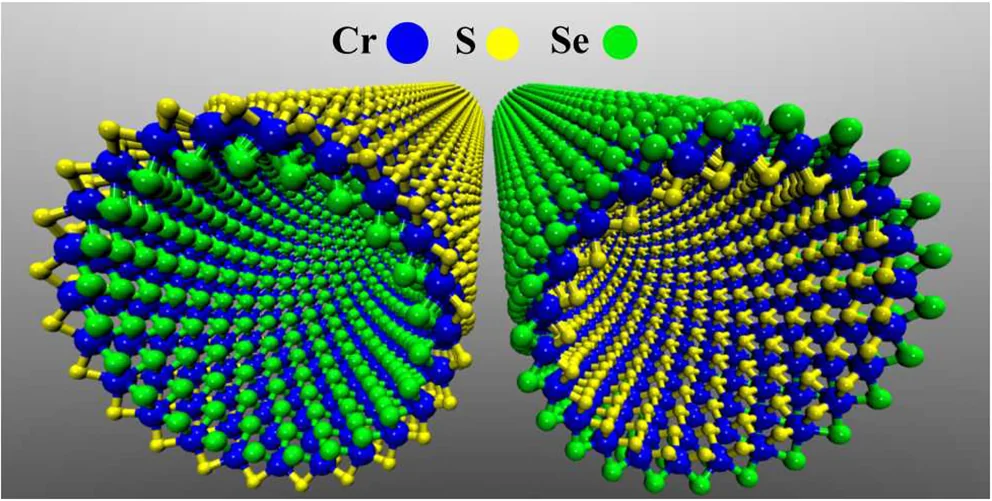

## Introduction

The development of clean and sustainable energy technologies is among the most pressing challenges of modern society. In this context, molecular hydrogen (H_2_) is a highly attractive energy carrier due to its high gravimetric energy density and carbon-free end-use. Electrochemical water splitting, and in particular the hydrogen evolution reaction (HER), offers a scalable route to green hydrogen production; however, its practical deployment hinges on electrocatalysts capable of reducing the overpotential and accelerating the reaction kinetics. Platinum (Pt) and Pt-based alloys remain the benchmark HER catalysts, yet their scarcity and high cost severely limit large-scale implementation, motivating the search for earth-abundant alternatives.^1–3^

Two-dimensional transition-metal dichalcogenides (2D-TMDs) have emerged as compelling candidates due to their chemical stability and broadly tunable electronic structure.^4–7^ Nevertheless, for conventional semiconducting TMDs such as MoS_2_ and WS_2_, HER activity is typically dominated by metallic edge sites, while the basal plane remains comparatively inert, imposing a fundamental limitation on performance.^4,6–8^ A promising route to overcome this constraint is offered by Janus-TMD monolayers (MXY), where distinct chalcogens occupy the top and bottom sublayers, breaking out-of-plane mirror symmetry.^9^ This structural asymmetry induces an intrinsic dipole moment and a built-in electric field, enabling band-structure engineering and charge redistribution that can be leveraged to activate catalytic sites.^8–10^ More broadly, recent studies on noncentrosymmetric 2D materials have shown that out-of-plane polarization can tune surface adsorption, charge transfer, reaction pathways, and even the competition with HER, highlighting the catalytic relevance of intrinsic electrostatic asymmetry.^11^

Within this family, Janus CrSSe has attracted growing attention as a mechanically robust and electronically versatile monolayer. Compared with its parent compounds (CrS_2_ and CrSe_2_), CrSSe combines mechanical and dynamical stability with attractive optoelectronic and piezoelectric responses, while retaining a semiconducting character that is sensitive to external perturbations.^10,12^ For HER, a central challenge is to activate and optimize basal-plane reactivity, which can be assessed through the hydrogen adsorption free energy, Δ*G*_ads_, a widely used descriptor where values near the thermo-neutral regime (Δ*G*_ads_ ≈ 0 eV) indicate favorable surface thermodynamics.^1–3^ Previous studies on Janus and related TMD systems have shown that chemical strategies such as vacancy engineering, hetero-atom doping, and single-atom adsorption can tune Δ*G*_ads_ toward thermo-neutrality by introducing electronic states near the Fermi level and modifying local charge density.^8,13–16^ For example, non-noble-metal doping has been shown to activate the basal plane of Janus VSSe by strengthening the orbital coupling between dopant p states and H-1s states.^16^ However, chemical functionalization typically requires careful control over defect concentrations and dopant distributions, which may complicate the synthesis and reproducibility.^8,13,14^

Beyond chemical modification, strain engineering and curvature provide powerful and reversible physical degrees of freedom to tailor catalytic properties, although their combined effect remains comparatively underexplored for CrSSe. Tensile strain can reduce the band gap and reorganize the electronic structure in Janus monolayers, potentially optimizing Δ*G*_ads_ without resorting to high defect densities.^12,17,18^ Previous studies have also shown that Janus MoSSe and MoSTe nanotubes exhibit diameter-, chirality-, outer-layer-, and strain-dependent electronic properties, including curvature-induced band gap modulation and possible semiconductor-to-metal transitions.^19,20^ Moreover, curvature has emerged as an effective strategy to modulate local bonding, orbital hybridization, charge transfer, and adsorption/desorption energetics in electrocatalytic systems.^21^ For instance, transition-metal-encapsulated carbon nanotubes have shown enhanced HER activity compared with analogous graphene-based systems, highlighting the role of nanotube curvature in tuning electronic interactions relevant to H adsorption.^22^ In addition, orientation-dependent studies along zigzag and armchair directions have demonstrated that structural directionality can influence electronic descriptors and catalytic activity.^23^ Despite these advances, the cooperative role of curvature and strain in activating basal-plane HER sites of Janus CrSSe nanotubes remains unexplored. In Janus systems, rolling a 2D sheet into a nanotube may further induce curvature-driven rehybridization, polarity-dependent charge redistribution, and electronic transitions relevant to adsorption thermodynamics.^10,17,24^

From an experimental perspective, the controlled synthesis of single-walled Janus CrSSe nanotubes has not yet been demonstrated and, therefore, remains an important challenge. Nevertheless, recent progress in one-dimensional TMD fabrication provides several plausible routes to such structures. TMD nanotubes have been obtained through template-assisted growth, metal-catalyzed chemical vapor deposition, and the spontaneous curling of flexible nanosheets, while curved Janus MoSSe and WSSe nanostructures have also been experimentally produced through surface chalcogen substitution, followed by self-curling.^24^ These developments, together with previous first-principles predictions identifying Cr-containing Janus-TMD nanotubes as stable configurations,^17^ support the experimental plausibility of extending similar approaches to CrSSe. In practice, however, nanotube formation may involve partial strain relaxation, vacancies, grain boundaries, or other curvature-induced defects, particularly for small diameters. Such imperfections could modify the local electronic structure and the H* adsorption energetics. The defect-free nanotubes considered here should therefore be regarded as idealized reference structures for isolating the intrinsic effects of curvature, Janus polarity, and axial strain.

Motivated by the mechanical robustness of Janus CrSSe, here we present a systematic first-principles (DFT) study of mechanochemical activation for HER, explicitly comparing H* adsorption on the S- and Se-terminated basal planes and tracking the associated band-structure/PDOS changes and charge redistribution. Unlike previous studies mainly focused on Mo-based Janus systems, defect-assisted HER activation, or electronic structure modulation alone, this work addresses pristine CrSSe and examines how uniaxial/biaxial strain and curvature, modeled through zigzag CrSSe-NT­(*n*,0) nanotubes with different diameters, cooperatively tune the binding energy and Δ*G*_ads_. This establishes direct links between deformation/curvature, orbital rehybridization, charge redistribution, and adsorption thermodynamics as a design guideline for activating basal-plane sites.

## Computational Methods

To systematically investigate mechanochemical activation (via strain and curvature) toward the hydrogen evolution reaction (HER), the Janus 2D CrSSe monolayer was selected as the reference system. As shown in Figure 1a, an orthorhombic (3 × 2 × 1) supercell was adopted, ensuring a lateral separation of ≃ 9.5 Å between periodically repeated adsorbates on the surface. Due to its intrinsic out-of-plane polarity, Janus CrSSe presents two nonequivalent basal surfaces: an S-terminated face, denoted as CrSSe-S, and a Se-terminated face, denoted as CrSSe-Se. Curvature effects were introduced by rolling the monolayer into CrSSe nanotubes.^25,26^ In this work, we focus on zigzag CrSSe-NT­(*n*,0) chiralities, as schematically illustrated in Figure 1b. The zigzag CrSSe-NT­(*n*,0) family was selected as a controlled model system to systematically tune curvature through the chiral index *n* while keeping comparable adsorption environments. This allows a direct correlation between diameter, electronic structure, and H* adsorption thermodynamics. Armchair and other chiralities may exhibit additional chirality-dependent effects and will be addressed in future work.

**Figure 1:**
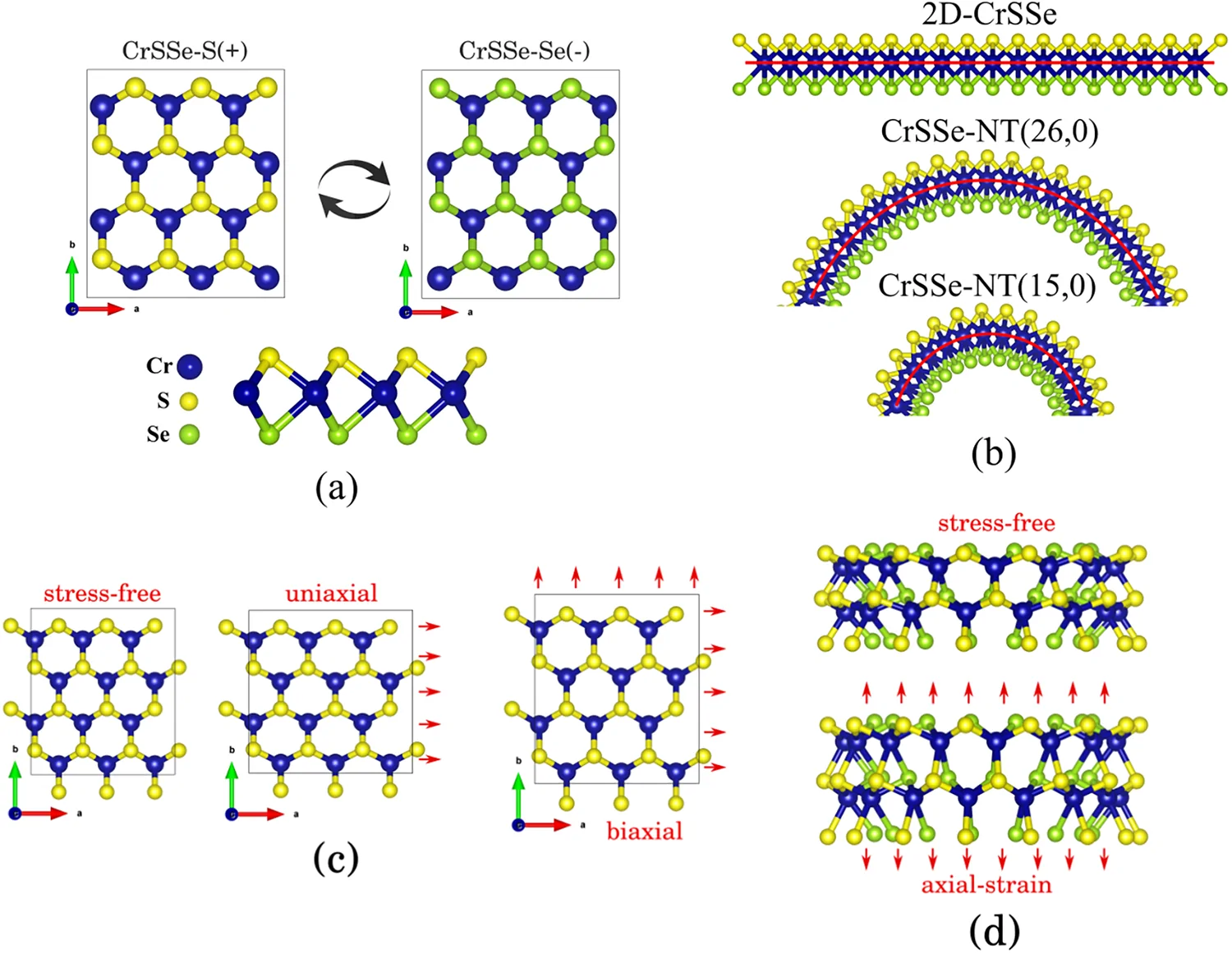
Atomic models and deformation protocols of Janus CrSSe. (a) Opposite CrSSe-S and CrSSe-Se orientations and side view of the monolayer (orthorhombic supercells 3 × 2). (b) Curvature engineering from planar CrSSe to representative (26,0) and (15,0) nanotubes. (c) Uniaxial and biaxial strain applied to 2D CrSSe. (d) Axial strain applied to CrSSe-NT-S­(15,0). Blue, yellow, and green spheres denote Cr, S, and Se atoms, respectively.

The zigzag CrSSe-NT­(*n*,0) structures were generated using the Cif2Tube software,^25^ which maps the Cartesian coordinates of the relaxed 2D CrSSe monolayer onto a cylindrical geometry. In this construction, the nanotube is defined by the chiral vector **C**_*h*_ = *n* **a**_1_ + *m* **a**_2_, where **a**_1_ and **a**_2_ are the in-plane lattice vectors. For the zigzag family considered here, *m* = 0, so that **C**_*h*_ = *n* **a**_1_ defines the nanotube circumference and the initial tube radius is given by R = |**C**_*h*_|/2π. The Cartesian-to-cylindrical coordinate transformation is given by^25–27^

1 $$\begin{array}{l} {x_{i}}^{\prime }=\left(R+sz_{i}\right)\cos\left(x_{i}/R\right),\\ {y_{i}}^{\prime }=\left(R+sz_{i}\right)\sin\left(x_{i}/R\right),\\ {z_{i}}^{\prime }=y_{i}, \end{array}$$

where *x*_*i*_ is the coordinate along the rolling direction, *y*_*i*_ is the coordinate along the nanotube axis, and *z*_*i*_ is the out-of-plane coordinate of the Janus monolayer. The parameter *s* = ±1 determines the rolling orientation and, consequently, whether the S- or Se-terminated Janus surface is exposed on the outer wall. All generated nanotube models were fully relaxed before the electronic structure and HER calculations. Accordingly, increasing *n* leads to larger tube diameters and therefore lower curvature, whereas smaller *n* yields smaller diameters and stronger curvature.

Finally, the strain protocols considered for both planar and curved structures are summarized in Figure 1c,d: for 2D CrSSe, in-plane uniaxial and biaxial deformations are applied, while for CrSSe nanotubes, uniaxial strain is applied along the tube axis. For clarity, biaxial strain was applied only to planar 2D CrSSe, while nanotubes were strained only uniaxially along the tube axis. A biaxial deformation in nanotubes would additionally involve circumferential/radial loading and therefore corresponds to a different mechanical condition.

All strained structures were fully relaxed before the electronic structure and HER calculations, and no bond breaking, structural collapse, or irreversible reconstruction was observed within the strain range considered. We note that this geometrical integrity after static relaxation does not by itself establish dynamical or finite-temperature stability. Nevertheless, previous first-principles stress–strain calculations for Janus CrSSe reported critical tensile strains of approximately 25% and 17% under uniaxial and biaxial loading, respectively, placing the maximum tensile strain considered here (+11%) below the reported mechanical limits.^12^ The highly compressed configurations are therefore used primarily to characterize the nonlinear electronic and adsorption response at large deformation rather than to imply independently verified thermal stability.

All calculations were carried out within density functional theory (DFT) as implemented in the SIESTA software.^28,29^ The exchange-correlation effects were described using the generalized gradient approximation of Perdew–Burke–Ernzerhof (GGA-PBE),^30^ and long-range dispersion interactions were accounted for through the Grimme D3 correction (DFT-D3).^31^ A real-space grid corresponding to a mesh cutoff of 350 Ry was employed, and the Kohn–Sham states were expanded in a numerical atomic-orbital basis set of split-valence double-*ζ* plus polarization quality (DZP).

Because of the partially filled Cr-3d states, spin polarization was explicitly included in the self-consistent DFT calculations. Collinear spin-polarized calculations were performed by allowing the spin-up and spin-down electronic densities to relax independently. Initial magnetic moments were assigned to the Cr atoms, and the magnetic state was subsequently determined to be self-consistent. The converged total and atom-resolved magnetic moments were monitored for the pristine, strained, curved, and H-adsorbed systems.

Structural relaxations were performed using the conjugate-gradient algorithm until the maximum residual force was below 0.025 eV/Å for both the 2D CrSSe monolayer and CrSSe nanotubes. Brillouin-zone sampling was set to a 5 × 5 × 1 Monkhorst–Pack mesh for the 2D system,^32^ while for the one-dimensionally periodic nanotubes a 1 × 1 × 12 grid was used along the tube axis. To avoid spurious interactions between periodic images, a large vacuum spacing was included in the nonperiodic directions (∼20 Å along the out-of-plane direction for the monolayer and ∼20 Å in the directions perpendicular to the nanotube axis). Because Janus CrSSe has an intrinsic out-of-plane dipole moment, dipole-correction tests were performed for the planar H-adsorbed systems. For the most stable site b, the corrected Δ*G*_ads_ values are 0.78 eV for CrSSe-S and 1.69 eV for CrSSe-Se, differing by only 0.05 and 0.03 eV from the uncorrected values in Table 1. Thus, dipole corrections do not affect the adsorption energy trends or the main conclusions. For nanotubes, the Janus polarity becomes radial, and spurious intertube interactions were minimized using large vacuum spacing perpendicular to the tube axis. Band structures were computed along the high-symmetry paths Γ–X–S−Γ for 2D CrSSe orthorhombic supercells (for the hexagonal pristine cell, the Γ–M–K−Γ path was used) and Γ–Z for the nanotubes.

**Table 1:** DFT-D3 Total Energies and the Corresponding H* Adsorption Energetics for 2D Janus CrSSe at the Two Nonequivalent Adsorption Configurations, Sites a and b^a^

site	*E*(*H)	*E*(*)	*E*(H_2_)	*E*(H)	*E*_b_	*E*_ads_	Δ*G*_ads_
S-Terminated CrSSe Surface							
site a	–72,263.59	–72,248.93	–31.45	–13.47	–1.17	1.06	1.30
site b	–72,264.06	–72,248.93	–31.45	–13.47	–1.64	0.59	0.83
Se-Terminated CrSSe Surface							
site a	–72,263.13	–72,248.93	–31.45	–13.47	–0.71	1.52	1.76
site b	–72,263.17	–72,248.93	–31.45	–13.47	–0.75	1.48	1.72

a The results are grouped according to the exposed Janus surface, namely, the S-terminated and Se-terminated CrSSe surfaces. Here, *E*(*H) and *E*(*) represent the total energies of the H-adsorbed and pristine CrSSe systems, respectively. All energies are given in eV.

To assess the thermodynamics of H* adsorption and its modulation by strain and curvature, we evaluated the Gibbs free energy of hydrogen adsorption, Δ*G*_ads_, and the H binding energy, *E*_b_. Within the computational hydrogen electrode (CHE) framework, Δ*G*_ads_ is defined as^33,34^

2 $$\Delta G_{\mathrm{ads}}=E_{\mathrm{ads}}^{1/2H_{2}}+\Delta E_{Z\mathrm{PE}}-T\Delta S$$

where the adsorption energy is^33,34^

3 $$E_{\mathrm{ads}}^{1/2H_{2}}=E_{\mathrm{CrSSe}+H}-\left(E_{\mathrm{CrSSe}}+\frac{1}{2}E_{H_{2}}\right)$$

Here, *E*_CrSSe+H_, *E*_CrSSe_, and $\frac{1}{2}E_{H_{2}}$ are the total DFT energies of the adsorbed system, the clean substrate (monolayer or nanotube), and half of the energy of an isolated H_2_ molecule, respectively. The zero-point energy (Δ*E*_*ZPE*_) contribution and the entropic correction (−*T*Δ*S*) were accounted for by adopting a constant value of 0.24 eV at *T* = 298.15 K, which is widely accepted in the HER literature.^27,35^ The H binding energy was computed as^33,34^

4 $$E_{b}=E_{\mathrm{CrSSe}+H}-\left(E_{\mathrm{CrSSe}}+E_{H}\right)$$

In the definition of the binding energy, we replaced the molecular reference term $\frac{1}{2}E\left(H_{2}\right)$ with the energy of an isolated gas-phase H atom, *E*_H_. With this convention, *E*_b_ directly quantifies the strength of the H–substrate interaction, whereas Δ*G*_ads_ remains the central HER descriptor; values of Δ*G*_ads_ close to 0 eV indicate thermo-neutral adsorption and, therefore, favorable HER adsorption thermodynamics.^27,33,34^

We emphasize that Δ*G*_ads_ is a thermodynamic descriptor of H* adsorption and does not by itself determine the kinetic activity or the rate-limiting HER step. A complete kinetic description would require explicit activation barriers for the Volmer, Heyrovsky, and/or Tafel pathways under electrochemical conditions, including electrode potential, solvent, interfacial electric field, and double-layer effects. Accordingly, the present study is restricted to the thermodynamic screening of mechanically induced changes in H* adsorption within the computational hydrogen electrode framework, without explicit aqueous electrolyte. Although these effects may influence the absolute Δ*G*_ads_ values, the relative trends induced by strain, curvature, and surface polarity remain meaningful because all systems were treated consistently within the same computational framework.

As shown in Figure 2a, two nonequivalent adsorption sites were considered to probe the catalytic response of both planar 2D CrSSe and CrSSe nanotubes with S- and Se-terminated outer surfaces (CrSSe-NT-S and CrSSe-NT-Se, respectively), which expose distinct chalcogen terminations depending on the tube polarity (see Figure 2b–e).

**Figure 2:**
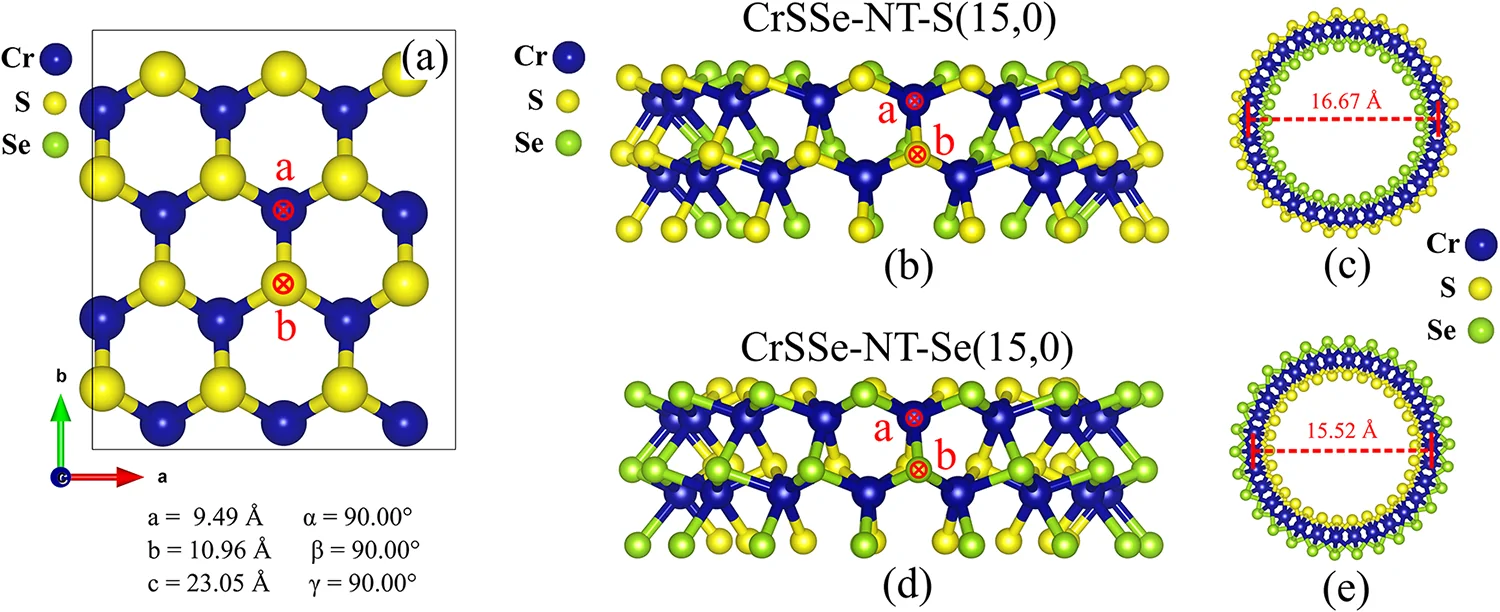
Hydrogen adsorption sites in planar and curved Janus CrSSe. (a) Top view of the CrSSe monolayer showing the nonequivalent H* adsorption sites *a* and *b*. (b, d) Side views of CrSSe-NT-S­(15,0) and CrSSe-NT-Se­(15,0) with the corresponding adsorption sites. (c, e) Cross-sectional views showing the nanotube diameters. Blue, yellow, and green spheres denote Cr, S, and Se atoms, respectively.

## Results and Discussion

Figure S1 summarizes the structural and electronic properties of pristine 2D Janus CrSSe. The relaxed conventional unit cell displays the characteristic bonding environment of the monolayer, with representative interatomic distances of Cr–Cr ≃ 3.12 Å, Cr–S ≃ 2.30 Å, and Cr–Se ≃ 2.45 Å, as well as an out-of-plane thickness of ≃3.08 Å (Figure S1a). The calculated electronic band structure along the high-symmetry path Γ–M–K−Γ reveals semiconducting behavior with an indirect band gap of 0.78 eV and no discernible spin splitting, with the Fermi level set to 0 eV (Figure S1b). The projected density of states (PDOS) further shows that the band-edge states are predominantly derived from Cr-3d orbitals, while S-3p and Se-4p contributions mainly hybridize away from the edges (Figure S1c). Overall, these results are consistent with previously reported data for Janus CrSSe and related Cr-based Janus systems,^10,12^ supporting the reliability of the present SIESTA-based description of 2D CrSSe.^36,37^

It is well established that the pristine basal planes of most semiconducting 2H-TMDs, and many Janus-TMDs, display intrinsically poor HER activity because the reaction largely proceeds at edge/defect sites rather than on the inert basal surface.^1,4,6,7^ In particular, the defect-free basal plane of MoS_2_ is predicted to exhibit strongly non-thermo-neutral adsorption, with adsorption free energy of Δ*G*_ads_ ≈ 2 eV, and approaching Δ*G*_ads_ ≈ 0 eV typically requires deliberate activation strategies such as introducing chalcogen vacancies and/or applying tensile strain, often in combination.^8,9^

A similar trend is obtained for 2D Janus CrSSe in the present study. For the CrSSe-S termination, the calculated H* binding energies are −1.17 eV and −1.64 eV at adsorption sites *a* and *b*, respectively, whereas for the CrSSe-Se termination, the corresponding values are −0.71 eV and −0.75 eV. The more negative binding energy at site *b* indicates a stronger H*–substrate interaction, consistent with the local adsorption geometry, where H binds preferentially to the chalcogen atom (S or Se) rather than to Cr, i.e., a more pronounced X–H (X = S, Se) interaction than a Cr–H interaction.

The electronic structure response to H* adsorption at the most stable site (site *b*) is shown in Figure 3a,c. In both CrSSe-S and CrSSe-Se, H* adsorption induces a clear shift in the band dispersion, driving the system toward metallic character as electronic states emerge at (or cross) the Fermi level. This behavior is further supported by the projected density of states in Figure 3b,d, which reveals a larger H-1s contribution in the valence-band region for CrSSe-S compared with CrSSe-Se. This enhanced H-1s hybridization is consistent with the stronger binding found for CrSSe-S­(+) (−1.64 eV) relative to CrSSe-Se (−0.75 eV). The trend is also reflected in the optimized bond lengths and charge redistribution: the shorter S–H and Se–H bonds (1.38 Å and 1.51 Å, respectively), together with the charge-density-difference maps in Figure 3e,f, indicate substantial charge rearrangement in the bonding region and a predominantly covalent character of the S–H and Se–H interactions. Such adsorption-induced electronic reorganization is consistent with previous reports showing that Janus asymmetry, vacancies, or adsorbate-induced states can shift the Fermi-level environment and modulate the HER activity of TMD-based catalysts.^8,13,14^

**Figure 3:**
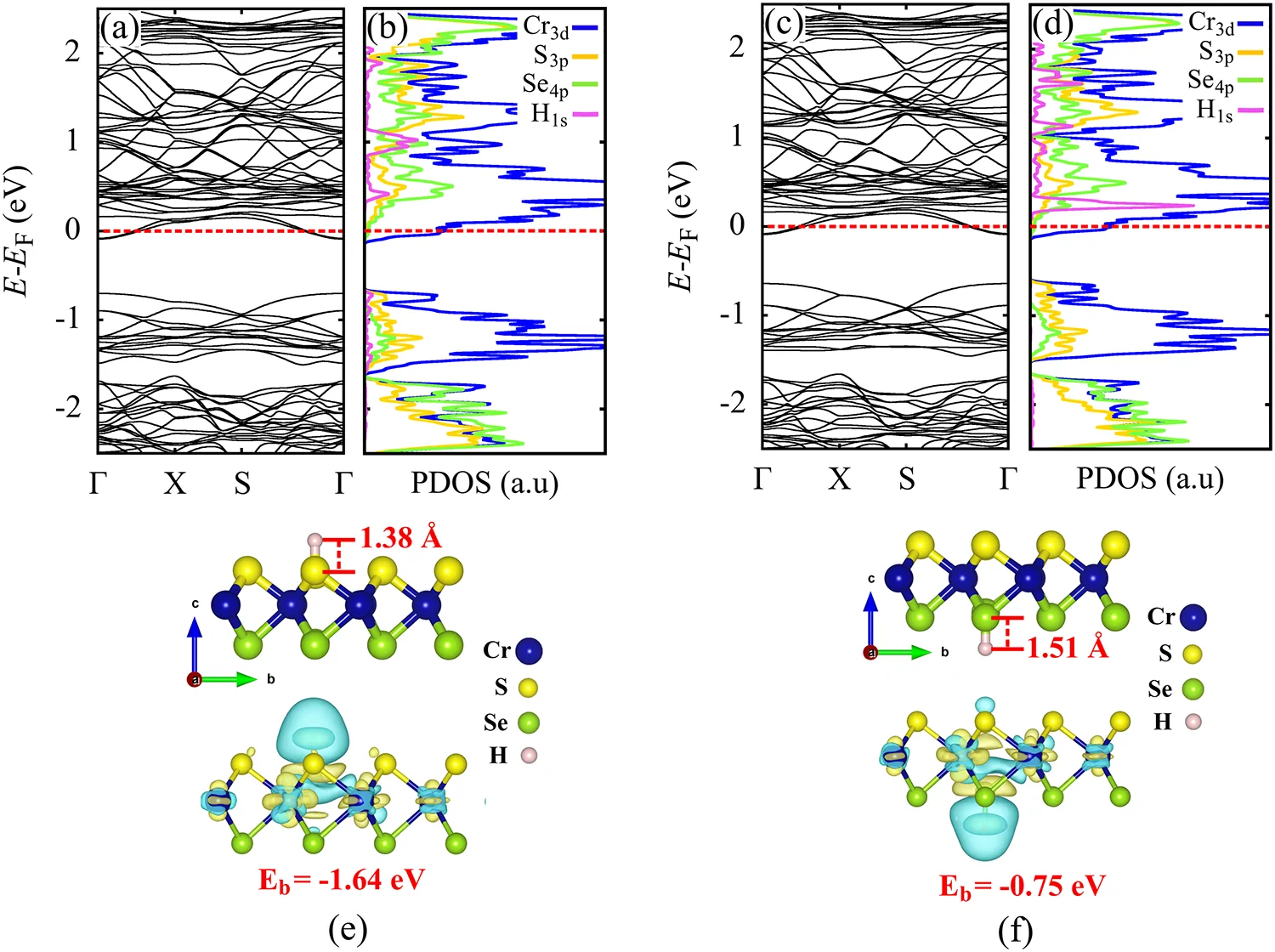
Electronic structure and charge redistribution upon H* adsorption on Janus CrSSe at site *b*. (a, b) Band structure and PDOS for the S-terminated surface and (c, d) for the Se-terminated surface. The red dashed line marks *E*_F_. (e, f) Relaxed adsorption geometries and charge-density difference maps, with the corresponding H–surface distances and binding energies *E*_b_.

Table 1 summarizes the H* binding energies *E*_b_, adsorption energies *E*_ads_, and adsorption free energies Δ*G*_ads_. Notably, the calculated Δ*G*_ads_ values are strongly positive, indicating a pronounced thermodynamic preference for H desorption and thus a clear lack of thermo-neutrality on pristine 2D CrSSe. This confirms that, despite its structural robustness and electronic tunability, pristine Janus CrSSe still requires an activation strategy to bring Δ*G*_ads_ toward the optimal HER window, in line with the broader behavior reported for semiconducting TMD and Janus-TMD basal planes.^1,4,6–8^

Given the intrinsically unfavorable H adsorption thermodynamics of pristine 2D CrSSe, it is necessary to explore viable activation strategies to enhance its catalytic performance. Figure 4 shows that in-plane mechanical deformation, applied either uniaxially or biaxially, substantially modifies the electronic band dispersion of Janus CrSSe, thereby tuning its band gap and the electronic states involved in H* adsorption. Representative band structures under compressive (−3%), unstrained (0%), and tensile (+3%) conditions along the *x* direction, *y* direction, and biaxially in the *xy* plane are presented in Figure 4a–c.

**Figure 4:**
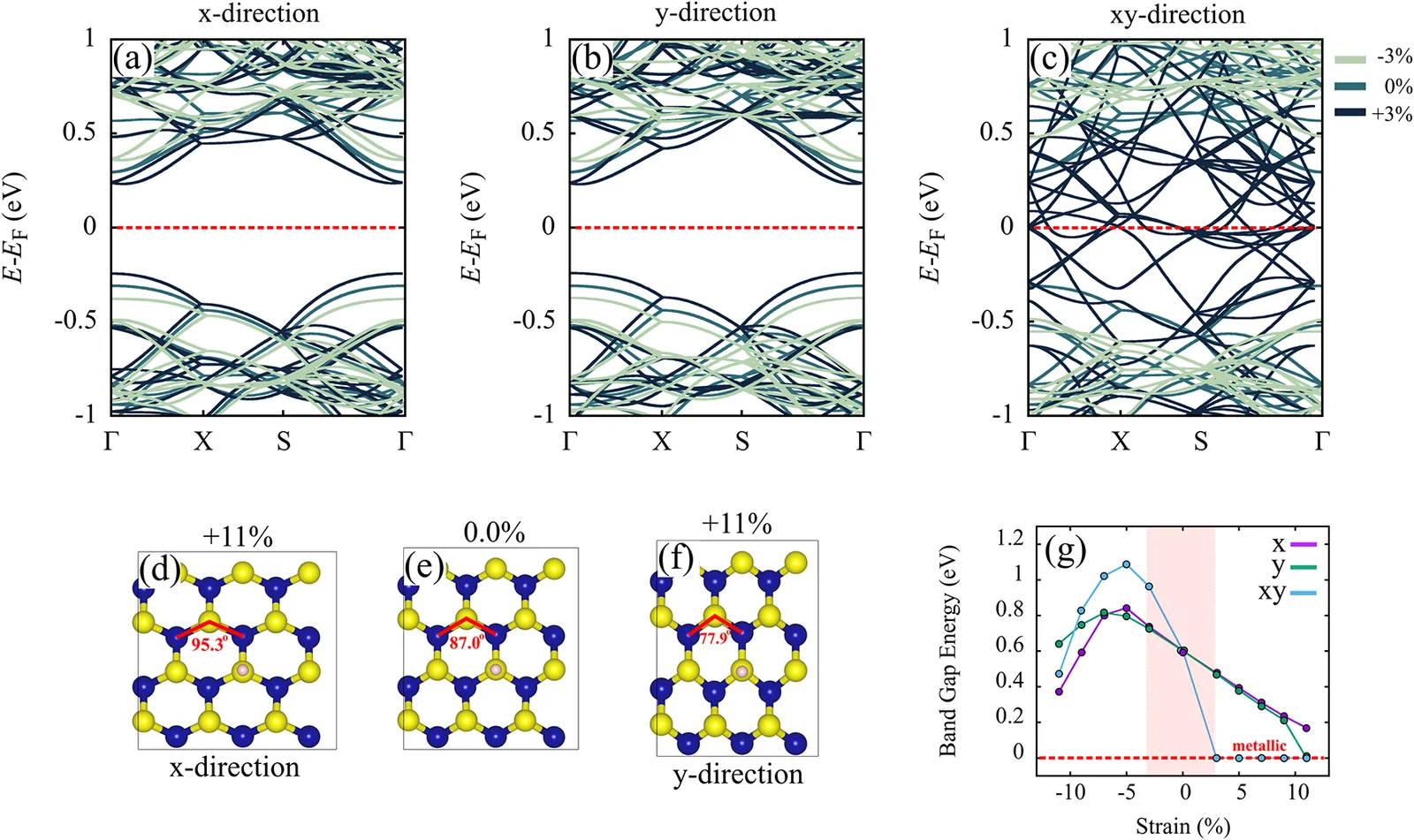
Strain-induced band-structure modulation in 2D Janus CrSSe. (a–c) Band structures under −3%, 0%, and +3% strain along the *x*, *y*, and biaxial *xy* directions. (d–f) Representative structures showing the strain-induced variation of the in-plane bond angle. (g) Band gap evolution with uniaxial and biaxial strain, including the strain-induced metallic regime.

As shown in Figure 4a,b, under uniaxial deformation, the band edges shift noticeably with strain, evidencing a clear electromechanical coupling between lattice distortion and electronic structure, in agreement with previous studies on Janus-TMD monolayers and related nanotubular systems.^12,17^ In both uniaxial directions, tensile strain progressively reduces the band gap, whereas compressive strain produces a comparatively moderate widening.

A more pronounced response is observed under biaxial deformation (Figure 4c). In particular, biaxial tensile strain drives the system toward a semiconductor-to-metal transition, which is already observed at approximately +3% strain, whereas biaxial compression produces a slight increase in the band gap. These results indicate that homogeneous in-plane strain provides an effective route for tuning the electronic character of 2D CrSSe, including the possibility of inducing a metallic state. Such strain-induced metallization may be advantageous for electrocatalytic applications, because enhanced electronic conductivity can facilitate charge transfer between the catalyst surface and adsorbed reaction intermediates.^5,6^

The microscopic origin of this behavior lies in the strain-induced distortion of the Cr–S and Cr–Se bonding network, which modifies orbital hybridization and redistributes the electronic states near the band edges. In addition, under specific strain conditions, these structural and electronic rearrangements can induce a spin-polarized electronic response, as observed for biaxially strained CrSSe.^12,17^ This interpretation is further supported by the S-3p/Cr-3d PDOS analysis in Figure S4a–c, which reveals strain-induced changes in both the spin polarization and intensity of the electronic states near the Fermi level.

The structural sensitivity becomes particularly evident at large tensile strain. As shown by the top-view snapshots in Figure 4d–f, a tensile deformation of +11% significantly distorts the pristine lattice and produces pronounced changes in the local bonding geometry. Specifically, the Se–Cr–S bond angle changes from approximately 87.0° in the relaxed structure to 95.3° under uniaxial loading along the *x* direction and to 77.9° under uniaxial loading along the *y* direction. These substantial angular distortions indicate that, at sufficiently large strains, the structural response enters a strongly nonlinear regime beyond small elastic deformations. Although these large-deformation configurations remain geometrically intact after relaxation, they should be distinguished from the moderate-strain regime and are not intended to establish finite-temperature stability. For tensile loading, however, the maximum strain explored here (+11%) remains below the previously reported critical tensile strains of Janus CrSSe (∼25% uniaxial and ∼17% biaxial).^12^

The overall evolution of the band gap as a function of strain, summarized in Figure 4g, further highlights the strong electromechanical tunability of Janus CrSSe. Under uniaxial loading along both the *x* and *y* directions, *E*_g_ varies approximately linearly within the small-strain regime (|ϵ| ≤ 5%), enabling relatively fine control of the electronic gap. Tensile uniaxial strain decreases *E*_g_ progressively, whereas compressive uniaxial strain tends to widen it. In contrast, the biaxial response is considerably stronger: tensile biaxial strain rapidly closes the gap and stabilizes a metallic electronic state from approximately +3% strain onward, whereas biaxial compression produces an approximately linear increase in *E*_g_ over the investigated range.

Beyond the small-strain regime, the electronic response becomes increasingly dependent on both the magnitude and mode of deformation, reflecting the nonlinear rearrangement of the Cr–S and Cr–Se bonding network. In particular, the metallic behavior induced by biaxial tension contrasts with the more gradual band gap reduction observed under uniaxial tensile loading. This qualitative behavior is consistent with previous first-principles studies showing that mechanical deformation can strongly modify the electronic properties of Janus CrSSe and related Janus-TMD nanotubes, including their band gaps and carrier effective masses, and can induce semiconductor-to-metal transitions through strain-driven orbital rehybridization.^12,17^

We note that GGA-PBE generally underestimates the absolute band gaps of semiconducting systems. Accordingly, the strain at which the gap closes in Figure 4g should be regarded as a PBE-level estimate, and the quantitative critical strain may shift under a higher-level exchange–correlation treatment such as HSE06. Nevertheless, previous PBE/HSE06 comparisons for Janus-TMD and CrSSe-containing systems indicate that hybrid functionals primarily increase the absolute band gap while preserving the qualitative electronic trends and band-edge character.^10,17^ Therefore, the PBE band structures are used here to describe the relative strain-induced electronic reconstruction rather than to provide a quantitatively exact transition threshold. Importantly, the HER trends discussed below are determined directly from the consistently calculated *E*_b_ and Δ*G*_ads_ values and do not rely on the absolute magnitude of the PBE band gap.

Figure 5a reveals a strongly deformation-mode-dependent magnetic response in 2D Janus CrSSe. The net spin magnetic moment remains essentially negligible throughout the investigated strain range for uniaxial deformation along the *x* direction. A similar non-spin-polarized response is obtained for *y*-directed strain up to +9%, followed by an abrupt increase to approximately 24 μ_B_/cell at +11%. In contrast, biaxial tensile strain induces a much earlier magnetic transition: a finite net spin moment emerges at approximately +3% and remains close to 24 μ_B_/cell up to +11%, whereas biaxial compression preserves an essentially vanishing net magnetization. This pronounced anisotropy demonstrates that the magnetic instability is governed not only by the strain magnitude but also by the symmetry of the imposed deformation. Notably, under biaxial tension, the onset of spin polarization occurs in the same strain range in which the semiconductor-to-metal transition is observed in Figure 4g, pointing to a common strain-driven reconstruction of the Cr-3*d*/chalcogen-*p* electronic states.

**Figure 5:**
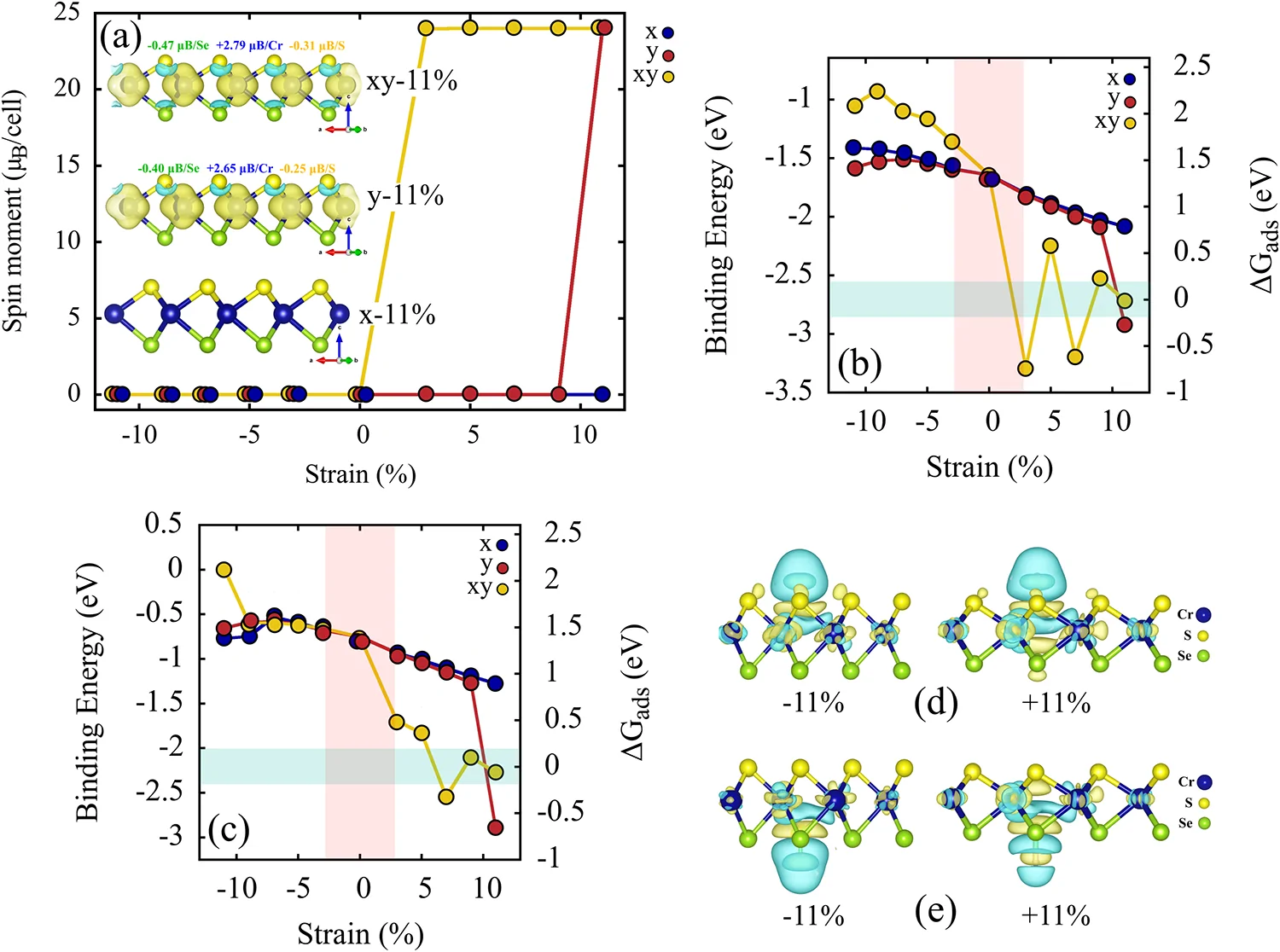
Strain-dependent magnetic response and hydrogen adsorption energetics of Janus CrSSe. (a) Net spin magnetic moment per cell as a function of uniaxial *x*, uniaxial *y*, and biaxial *xy* strain; representative spin-density isosurfaces (δ = 10^–2^ e/Å^3^) are shown for the indicated strain states. (b, c) H* binding energy (left axis) and adsorption free energy, Δ*G*_ads_ (right axis), at the most stable adsorption site *b* for the S- and Se-terminated surfaces, respectively. The vertical shaded region highlights the small-strain interval from −3% to +3%, while the horizontal shaded region denotes the near-thermo-neutral adsorption regime around Δ*G*_ads_ = 0 eV. (d, e) Charge-density difference isosurfaces (δ = 10^–3^ e/Å^3^) under biaxial −11% and +11% strain for H* adsorption on the S- and Se-terminated surfaces, respectively, illustrating the strain-induced redistribution of charge around the adsorption site.

The consequences of this electronic reconstruction for hydrogen adsorption are summarized in Figure 5b–e, where the H* binding energy and adsorption free energy, Δ*G*_ads_, are shown as functions of uniaxial and biaxial strain for the most stable adsorption configuration (site *b*). Because optimal HER thermodynamics require Δ*G*_ads_ close to zero, strain-induced strengthening of the H*–surface interaction is beneficial only until the thermo-neutral regime is approached; excessively negative values instead indicate H* overbinding and may hinder its subsequent desorption. The pronounced strain sensitivity observed here is consistent with previous first-principles studies showing that lattice deformation in Janus and related TMD systems modifies orbital hybridization, electronic-state alignment, charge redistribution, and ultimately hydrogen adsorption thermodynamics.^8,15,17^ The strong structural response of Janus CrSSe to mechanical deformation has likewise been established previously.^12^

For the S-terminated surface (Figure 5b), pristine CrSSe exhibits unfavorable hydrogen adsorption, with Δ*G*_ads_ ≈ 0.83 eV at zero strain. Uniaxial tensile deformation progressively lowers Δ*G*_ads_, indicating a gradual stabilization of adsorbed H*, whereas compression shifts Δ*G*_ads_ toward larger positive values and therefore weakens adsorption. Along the *x* direction, Δ*G*_ads_ decreases continuously to approximately 0.39 eV at +11%. A comparable evolution occurs under *y*-directed tension up to +9%, followed by an abrupt change to Δ*G*_ads_ ≈−0.44 eV at +11%. Interestingly, this discontinuous strengthening of H* adsorption coincides with the onset of the large spin magnetic moment observed in Figure 5a, indicating a substantial electronic reconstruction at this strain.

An even stronger response is produced by biaxial tensile deformation. Upon entering the spin-polarized metallic regime, Δ*G*_ads_ becomes strongly nonmonotonic, changing from approximately −0.81 eV at +3% to +0.24 eV at +5%, −0.72 eV at +7%, and finally approaching thermo-neutral adsorption at +9% (Δ*G*_ads_ ≈−0.05 eV). At +11%, adsorption remains favorable but becomes somewhat stronger, with Δ*G*_ads_ ≈ −0.24 eV. Importantly, the oscillatory evolution of Δ*G*_ads_ despite the nearly saturated spin moment over this strain interval indicates that spin polarization alone does not determine the catalytic response. Instead, the adsorption energetics reflect a coupled magneto-electronic and structural reconstruction involving changes in local bonding, orbital hybridization, and charge transfer at the adsorption site.

The Se-terminated surface (Figure 5c) displays a qualitatively similar but quantitatively distinct response. In the unstrained configuration, Δ*G*_ads_ ≈ 1.72 eV, demonstrating that H* adsorption on this termination is substantially less favorable than on the S side. Uniaxial tensile strain along *x* progressively decreases Δ*G*_ads_, although it remains far from thermo-neutral even at +11% (∼1.21 eV). The response along *y* follows a comparable trend up to +9%, but at +11% the adsorption free energy abruptly decreases to approximately −0.41 eV. As for the S termination, this discontinuity coincides with the strain-induced appearance of a large net spin moment, further supporting a strong coupling between the magnetic/electronic reconstruction and H* chemisorption.

Biaxial tension is particularly effective in activating the otherwise weakly reactive Se-terminated surface. Δ*G*_ads_ decreases sharply from 1.72 eV in the pristine system to approximately 0.78 and 0.65 eV at +3% and +5%, respectively, and reaches a nearly ideal value of Δ*G*_ads_ ≈ −0.07 eV at +7%. Further deformation produces a nonmonotonic response, with Δ*G*_ads_ ≈ 0.38 eV at +9% and ∼0.21 eV at +11%. In contrast, biaxial compression drives the adsorption free energy toward increasingly positive values, moving the system further away from the thermo-neutral condition. These results identify tensile biaxial deformation as the most effective mechanical route investigated here for driving the H adsorption thermodynamics of both CrSSe terminations toward the favorable HER regime.^8,15^

The charge-density-difference distributions in Figure 5d,e provide a microscopic picture of this strain-controlled chemisorption. The markedly different spatial redistribution of charge around the adsorbed H atom and the neighboring Cr–S/Cr–Se bonding network under −11% and +11% biaxial strain demonstrates that mechanical deformation substantially reorganizes the local electronic environment of the adsorption site. This redistribution is particularly relevant under tensile conditions, where the simultaneous structural distortion, electronic metallization, and emergence of spin polarization coincide with large changes in the H* binding thermodynamics. Taken together, these results reveal a coupled strain–magnetism–chemisorption mechanism in 2D Janus CrSSe, through which mechanical deformation can transform an intrinsically poor H*-binding surface into a regime approaching thermo-neutral hydrogen adsorption.^12,15,17^

Complementing the charge-density analysis, the spin-resolved S-3p and Cr-3d PDOS of pristine and biaxially strained 2D CrSSe are shown in Figure S4a–c. Pristine and −5% compressed CrSSe exhibit negligible states near the Fermi level and no appreciable spin polarization, consistent with their semiconducting character. In contrast, +5% biaxial tension shifts additional Cr-3d and S-3p states toward *E*_F_ and induces a marked spin polarization, evidencing a strain-driven electronic reconstruction. This coupled modification of orbital hybridization and spin-resolved states alters the H*–surface interaction and is consistent with the observed improvement in Δ*G*_ads_.^8,17^

Beyond biaxial strain, curvature offers another mechanically driven route to tune the electronic structure and the HER thermodynamics. Rolling the Janus monolayer into nanotubes introduces intrinsic curvature that can strongly modify bonding, band structure, and adsorption energetics.^17,24,38^

The curvature-induced magnetic response of zigzag Janus CrSSe nanotubes is shown in Figure 6a. A pronounced dependence on the nanotube orientation is observed. CrSSe-NT-S exhibits a finite spin polarization throughout the investigated range, whereas CrSSe-NT-Se converges to a zero-net-spin solution. For CrSSe-NT-S, the average magnetic moment remains nearly constant at ∼0.83 μ_B_/atom for 15 ≤ *n* ≤ 20, followed by an abrupt decrease to ∼0.66 μ_B_/atom at *n* = 21, after which it remains almost unchanged. This behavior reveals a curvature-driven crossover between two distinct magnetic regimes.

**Figure 6:**
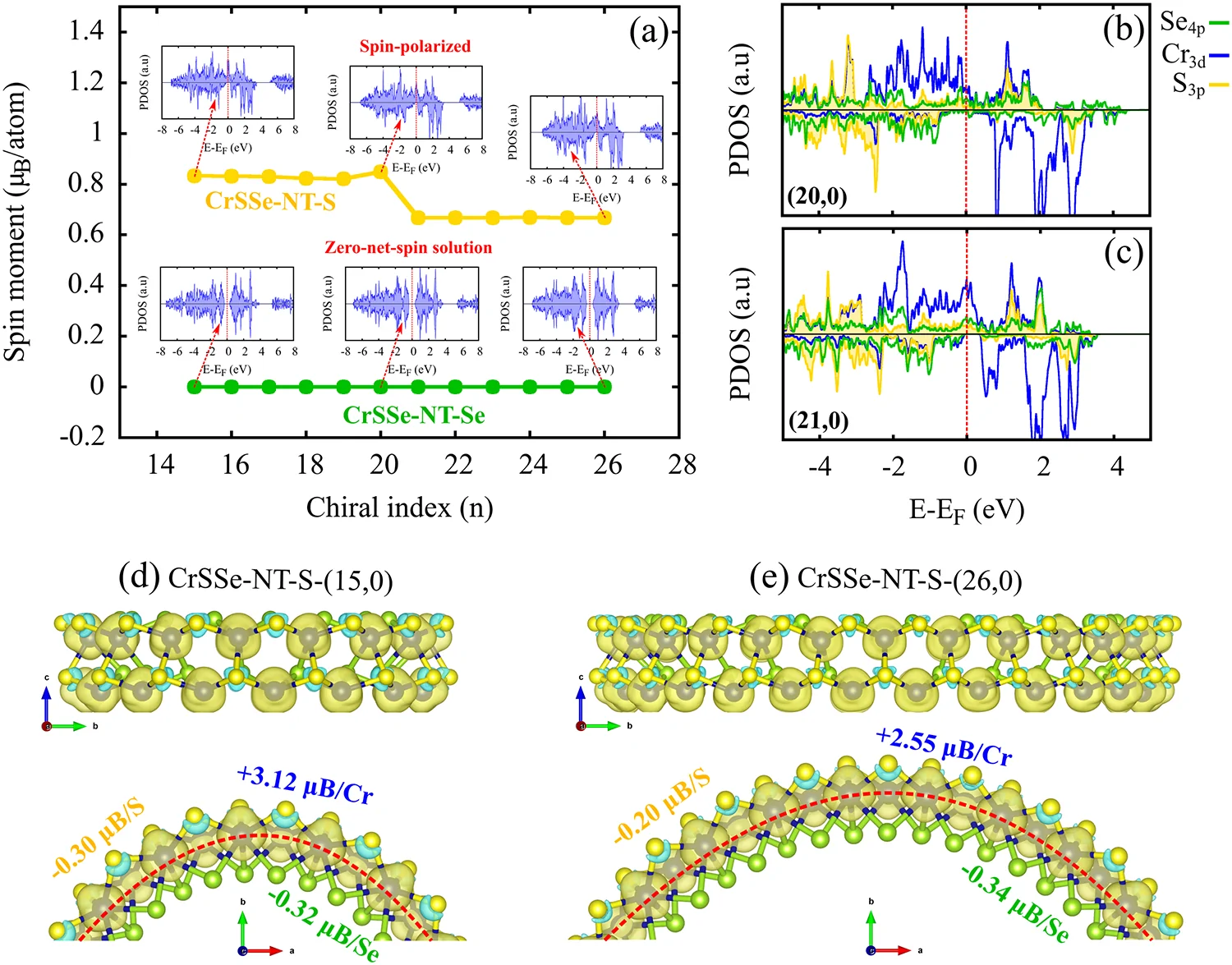
Curvature-dependent magnetic and electronic properties of zigzag Janus CrSSe nanotubes. (a) Average spin magnetic moment per atom as a function of the chiral index *n* for CrSSe-NT-S and CrSSe-NT-Se, together with representative spin-resolved PDOS for selected nanotubes. (b, c) Orbital-resolved spin-polarized PDOS of CrSSe-NT-S­(20,0) and CrSSe-NT-S­(21,0), showing the Se-4p, Cr-3d, and S-3p contributions. (d, e) Spin-density isosurfaces (δ = 10^–3^ e/Å^3^) for CrSSe-NT-S­(15,0) and CrSSe-NT-S­(26,0), respectively, highlighting the dominant positive local moments on Cr and the smaller antiparallel contributions on S and Se atoms.

The spin-resolved PDOS of the representative (20,0) and (21,0) nanotubes (Figure 6b,c) further shows that this transition is accompanied by a redistribution of the Cr-3d, S-3p, and Se-4p states, particularly near the Fermi level. The pronounced asymmetry between the two spin channels confirms that the magnetic response is primarily associated with the Cr-3d states and is modulated by curvature-dependent p–d hybridization.

This picture is reinforced by the spin-density distributions in Figure 6d,e. The dominant positive magnetic moment is localized on Cr, decreasing from approximately +3.12 μ_B_/Cr for CrSSe-NT-S­(15,0) to +2.55 μ_B_/Cr for CrSSe-NT-S­(26,0), while S and Se exhibit smaller antiparallel contributions. These results demonstrate that curvature and Janus orientation jointly control the magnetic state of CrSSe nanotubes through modification of the local Cr–chalcogen hybridization.

The curvature energy was evaluated as *E*_curv_ = *E*_NT_^CrSSe^ – *E*_2D_^CrSSe^, where *E*_NT_ is the total energy per atom of the nanotube and *E*_2D_^CrSSe^ is the energy per atom of 2D CrSSe. This quantity represents the energetic cost associated with rolling the 2D CrSSe sheet into a tubular geometry: larger *E*_curv_ values indicate a higher energetic penalty for curvature formation, whereas smaller *E*_curv_ values imply that the tubular structure is comparatively more favorable, as generally expected for low-dimensional nanotubular systems.^17,24,26,39^

Figure 7a shows curvature energy, *E*_curv_, as a function of the zigzag chiral index *n*. CrSSe-NT-Se exhibits systematically lower curvature energies than CrSSe-NT-S, particularly at small diameters, indicating that the configuration with Se atoms on the outer surface is energetically more favorable upon rolling. For both nanotube orientations, *E*_curv_ decreases toward the planar limit with an increase in *n*, consistent with the progressive reduction of curvature.^17,24,38,39^ It should be noted that the present nanotubes correspond to ideal defect-free structures. Full structural relaxation allows the curvature-induced bond lengths and lattice parameters to redistribute and therefore captures intrinsic elastic strain relaxation within an intact nanotube; however, it does not account for defect-mediated relaxation through vacancy formation, grain boundaries, or local discontinuities. Such defects may become increasingly relevant at high curvatures and could introduce additional active sites or modify the adsorption energetics in experimentally realized nanotubes.

**Figure 7:**
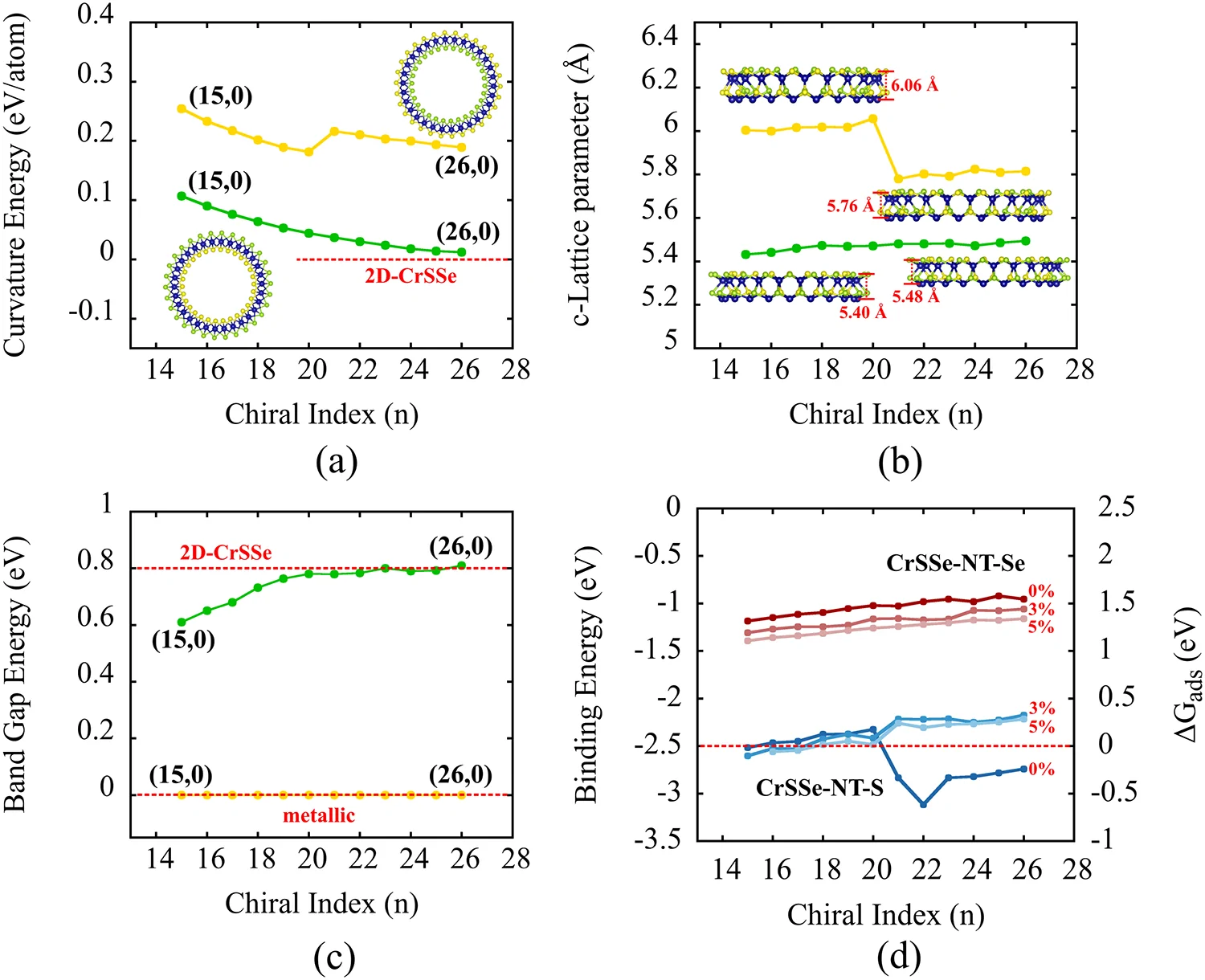
Curvature-dependent structural, electronic, and hydrogen adsorption properties of zigzag Janus CrSSe nanotubes. (a) Curvature energy as a function of the chiral index *n* for CrSSe-NT-S and CrSSe-NT-Se. (b) Axial lattice parameter *c*, highlighting the abrupt structural reconstruction of CrSSe-NT-S between (20,0) and (21,0). (c) Band gap versus *n*, showing semiconducting behavior for CrSSe-NT-Se and metallic behavior for CrSSe-NT-S throughout the investigated range. (d) H* binding energy and adsorption free energy, Δ*G*_ads_, under 0%, 3%, and 5% axial tensile strain. Red dashed lines indicate the corresponding 2D CrSSe reference values or the thermo-neutral condition Δ*G*_ads_ = 0.

A notable anomaly appears for CrSSe-NT-S between the (20,0) and (21,0) nanotubes. As shown in Figure 7b, the axial lattice parameter abruptly decreases from approximately 6.06 to 5.78 Å, whereas CrSSe-NT-Se evolves smoothly over the same diameter range. This structural reconstruction coincides with the abrupt reduction of the average spin moment observed in Figure 6a, from ∼0.83 to ∼0.66 μ_B_/atom. The simultaneous changes in axial periodicity and magnetization therefore indicate a curvature-driven crossover between two distinct structural–magnetic regimes of CrSSe-NT-S.

The corresponding electronic response is summarized in Figures 7c and S2. CrSSe-NT-Se remains semiconducting throughout the investigated range, with *E*_g_ increasing from approximately 0.61 eV for (15,0) to ∼0.81 eV for the largest nanotubes, approaching the 2D CrSSe value as curvature decreases. In contrast, CrSSe-NT-S remains metallic for all considered chiral indices, with spin-polarized bands crossing the Fermi level (Figure S2a–c). Thus, curvature produces a strongly orientation-dependent electronic response: placing S on the outer surface stabilizes a spin-polarized metallic state, whereas the opposite Janus orientation preserves semiconducting behavior.

These results demonstrate that the electronic and magnetic properties of CrSSe nanotubes are governed not only by diameter, but also by the orientation of the intrinsic Janus asymmetry. In particular, the pronounced structural reconstruction at *n* = 20 → 21 in CrSSe-NT-S provides a direct link between curvature-induced lattice relaxation and the magnetic crossover identified in Figure 6. Such strong curvature sensitivity is consistent with previous first-principles studies showing that orbital rehybridization in Janus-TMD nanotubes can substantially modify their band structure and magnetic/electronic response.^17,38^

Figure S3 further clarifies the electronic origin of the polarity-dependent H* adsorption in CrSSe nanotubes. CrSSe-NT-S­(15,0) preserves its metallic character after adsorption and exhibits a pronounced H-1s contribution near the Fermi-level region, together with Cr-3d and S-3p states, consistent with strong electronic coupling between H* and the S-terminated outer wall. In contrast, H adsorption on CrSSe-NT-Se­(15,0) produces a weaker H-1*s* contribution and a redistribution of the electronic states toward the band-edge region, while retaining a substantially depleted density of states around *E*_F_. The corresponding charge-density-difference maps (Figure S3c,f) further evidence termination-dependent charge rearrangement around the H–chalcogen bonding region. These results are consistent with the stronger H* binding obtained for the S-terminated nanotubes and with previous reports showing that adsorption-induced states can substantially modify the electronic structure of Janus-TMD catalysts.^8,13,14^

The influence of curvature on the electronic structure is also evident from the S-3*p*/Cr-3*d* PDOS in Figure S4d,e. The smaller CrSSe-NT-S­(15,0) nanotube exhibits a stronger redistribution of states near *E*_F_ than CrSSe-NT-S­(26,0), indicating that increased curvature modifies the local Cr–S p–d hybridization. This curvature-induced reconstruction provides an electronic basis for the pronounced diameter dependence of H* adsorption.

The corresponding HER energetics are summarized in Figure 7d. For CrSSe-NT-Se, decreasing the nanotube diameter generally strengthens H* adsorption and lowers Δ*G*_ads_, although the adsorption remains considerably less favorable than for the S-terminated nanotubes. In contrast, CrSSe-NT-S exhibits a distinctly nonmonotonic response. From *n* = 15 to *n* = 20, decreasing curvature progressively weakens H* adsorption; however, an abrupt strengthening occurs between (20,0) and (21,0), producing strongly negative Δ*G*_ads_ values around *n* = 21–22. Remarkably, this anomaly occurs at the same chiral index at which the axial lattice parameter drops abruptly (Figure 7b) and the average spin moment decreases (Figure 6a). The simultaneous structural, magnetic, and adsorption changes therefore indicate that the curvature-driven crossover identified above also directly controls the H* chemisorption strength.

This result also emphasizes that stronger adsorption does not necessarily imply improved HER thermodynamics. While curvature can drive Δ*G*_ads_ toward the thermo-neutral condition, the pronounced strengthening around *n* = 21–22 in unstrained CrSSe-NT-S leads instead to H* overbinding. Consequently, the catalytic response is governed by an interplay between curvature, Janus polarity, and the associated structural–magnetic reconstruction, rather than by nanotube diameter alone.

Axial tensile strain provides an additional degree of control over this curvature-dependent response. As shown in Figure 7d, applying 3% and 5% strain substantially reshapes the adsorption energy curves, particularly for CrSSe-NT-S, and suppresses the pronounced adsorption anomaly observed near the structural–magnetic crossover. The strained S-terminated nanotubes maintain Δ*G*_ads_ much closer to the thermo-neutral regime over a broad range of chiral indices, whereas the Se-terminated systems show only a moderate reduction and remain comparatively weak H* binders. Among the strains considered, 5% tensile deformation produces the strongest overall stabilization of adsorbed H*; however, the optimum HER condition remains chirality-dependent because excessively strong binding can shift Δ*G*_ads_ below zero.

The microscopic effect of axial strain is further supported by Figure S4f. Relative to the unstrained CrSSe-NT-S­(15,0) nanotube, +5% axial tension markedly redistributes the Cr-3d and S-3p spectral weight near *E*_F_, evidencing an additional modification of the local p–d hybridization. This electronic reconstruction is consistent with the strain-induced changes in H* binding shown in Figure 7d. Overall, the results reveal that curvature and axial strain are not independent perturbations, but jointly regulate the local bonding, magnetic state, and adsorption thermodynamics of CrSSe nanotubes.^15,17^

As summarized in Table 2, mechanical activation drives CrSSe into the same near-thermo-neutral H* adsorption regime reported for several state-of-the-art TMD-based HER catalysts. In particular, biaxially strained CrSSe-S and CrSSe-Se reach Δ*G*_ads_ = −0.05 and −0.07 eV, respectively, comparable to active MoS_2_/WS_2_ edge sites and to vacancy- or single-atom-engineered Janus systems. An even more favorable thermodynamic condition is obtained for CrSSe-NT-S­(20,0) under +5% axial strain, for which Δ*G*_ads_ = −0.01 eV, essentially satisfying the ideal thermo-neutral criterion. Remarkably, this value is achieved without introducing vacancies, dopants, or foreign single-atom centers, highlighting the effectiveness of combining curvature and strain as a chemically noninvasive activation strategy. In contrast, the best CrSSe-NT-Se configuration remains far from thermo-neutrality (Δ*G*_ads_ = 1.083 eV), demonstrating that curvature alone does not guarantee favorable H* adsorption and that the orientation of the intrinsic Janus polarity plays a decisive role in determining the catalytic response. Nevertheless, these comparisons should be interpreted strictly in terms of H* adsorption thermodynamics, since experimental HER performance also depends on reaction kinetics, charge transport, electrolyte conditions, active-site density, and long-term stability.

**Table 2:** Representative Theoretical and Experimental HER Benchmarks Compared with Mechanically Activated Janus CrSSe^a^

system	activation strategy	Δ*G*_H*_ (eV)	experimental HER metric	ref
Pt/C	benchmark catalyst	∼−0.09	η_10_ ∼ 46 mV; ∼44.3 mV dec^–1^	^40^
MoS_2_	active edge	∼0.06–0.08		^2,5^
1T-WS_2_	phase engineering	∼0.28	onset ∼80–100 mV; ∼55 mV dec^–1^	^5,7^
1T-MoSSe nanodots	phase/size engineering	∼0.19	η_10_ ∼ 140 mV; ∼40 mV dec^–1^	^1^
Se-vacancy WSSe	vacancy engineering	∼0.02		^8^
2H-*V*_Cr_-CrSTe	vacancy engineering	0.03		^13^
1T-*V*_Cr_-CrSeTe	vacancy engineering	–0.02		^13^
Ru@CrSSe	single-atom engineering	–0.07		^14^
CrSSe-S, +9% biaxial	biaxial strain	–**0.05**		this work
CrSSe-Se, +7% biaxial	biaxial strain	–**0.07**		this work
CrSSe-NT-S(20,0), +5% axial	curvature + axial strain	–**0.01**		this work
CrSSe-NT-Se(15,0), +5% axial	curvature + axial strain	+1.08		this work

a Literature Δ*G*_H*_ values are compared with the Δ*G*_ads_ values obtained in this work, whereas experimental overpotentials and Tafel slopes are provided as complementary practical benchmarks and should not be interpreted as quantities directly equivalent to adsorption free energies.

## Conclusions

In this work, we systematically investigated the mechanochemical activation of Janus CrSSe toward the hydrogen evolution reaction using spin-polarized first-principles calculations. Pristine 2D CrSSe is an indirect-gap semiconductor (*E*_g_ ≈ 0.78 eV) with intrinsically unfavorable basal-plane H* adsorption and a pronounced polarity dependence, yielding Δ*G*_ads_ ≈ 0.83 and 1.72 eV for the S- and Se-terminated surfaces, respectively. Mechanical deformation strongly modifies this response by reorganizing the Cr-3d/chalcogen-p electronic states. In particular, biaxial tensile strain induces a semiconductor-to-metal transition and the onset of spin polarization at approximately +3%, accompanied by substantial changes in H* binding. Nearly thermo-neutral adsorption is achieved at selected tensile strains, with Δ*G*_ads_ ≈ −0.05 eV for the S termination at +9% and ≈−0.07 eV for the Se termination at +7%. These results reveal that the catalytic response arises from a coupled structural, orbital, and spin-dependent electronic reconstruction rather than from strain-induced magnetization alone.

Curvature introduces an additional and strongly polarity-dependent degree of freedom. Zigzag CrSSe-NT-S nanotubes exhibit spin-polarized metallic behavior throughout the investigated diameter range, whereas CrSSe-NT-Se retains essentially zero net spin and remains semiconducting, progressively approaching the electronic structure of the planar monolayer as curvature decreases. Particularly noteworthy is the abrupt crossover between CrSSe-NT-S­(20,0) and (21,0), where simultaneous changes in the axial lattice parameter, magnetic moment, and H* binding identify a direct coupling among curvature-induced structural relaxation, magnetism, and chemisorption. Thus, the HER response of CrSSe nanotubes cannot be described solely in terms of nanotube diameter but instead reflects the interplay between curvature and the orientation of the intrinsic Janus polarity.

Finally, moderate axial tensile strain provides a complementary means of controlling this curvature-dependent adsorption response. Strains of 3–5% substantially reshape the H* adsorption energetics, particularly for the S-terminated nanotubes, and can suppress the strong overbinding associated with the structural–magnetic crossover while maintaining Δ*G*_ads_ closer to the thermo-neutral regime over a broad range of chiral indices. The corresponding redistribution of Cr-3d and S-3p states near the Fermi level confirms that strain and curvature jointly regulate local orbital hybridization and adsorption thermodynamics. Overall, our results establish Janus polarity, strain, and curvature as interconnected physical descriptors for controlling the electronic, magnetic, and H* adsorption properties of CrSSe, providing a chemically noninvasive strategy for engineering basal-plane H* adsorption in Janus-TMD nanostructures.

## Supplementary Material



## Notes

Structural/electronic properties of pristine 2D Janus CrSSe, electronic band structures of CrSSe nanotubes, charge redistribution upon H* adsorption, and S-3p/Cr-3d PDOS analysis under strain and curvature; optimized Cartesian coordinates of representative 2D CrSSe, CrSSe-NT-S­(15,0), and CrSSe-NT-Se­(15,0) structures in VASP format (PDF)
