# Supplementary material for: Modulating the Electronic Structure and Basal-Plane Adsorption of Janus CrSSe through Mechanochemical Activation
Source: ACS Omega. 2026 Oct 7;11(41):62744–55. doi: 10.1021/acsomega.6c04409 (PMC13689254; doi:10.1021/acsomega.6c04409)
Supplement: Supplementary file 1 [file ao6c04409_si_001.pdf]

SUPPLEMENTARY MATERIAL: Modulating the Electronic Structure and Basal-Plane Adsorption of Janus CrSSe through Mechanochemical Activation

Juan Gomez Quispe and Pedro Alves da Silva Autreto*

Center of Natural and Human Sciences, Federal University of ABC, Santo Andre, Sao Paulo, Brazil

E-mail: pedro.autreto@ufabc.edu.br

Phone: +55 (19) 98279-4988

Supporting Information Available

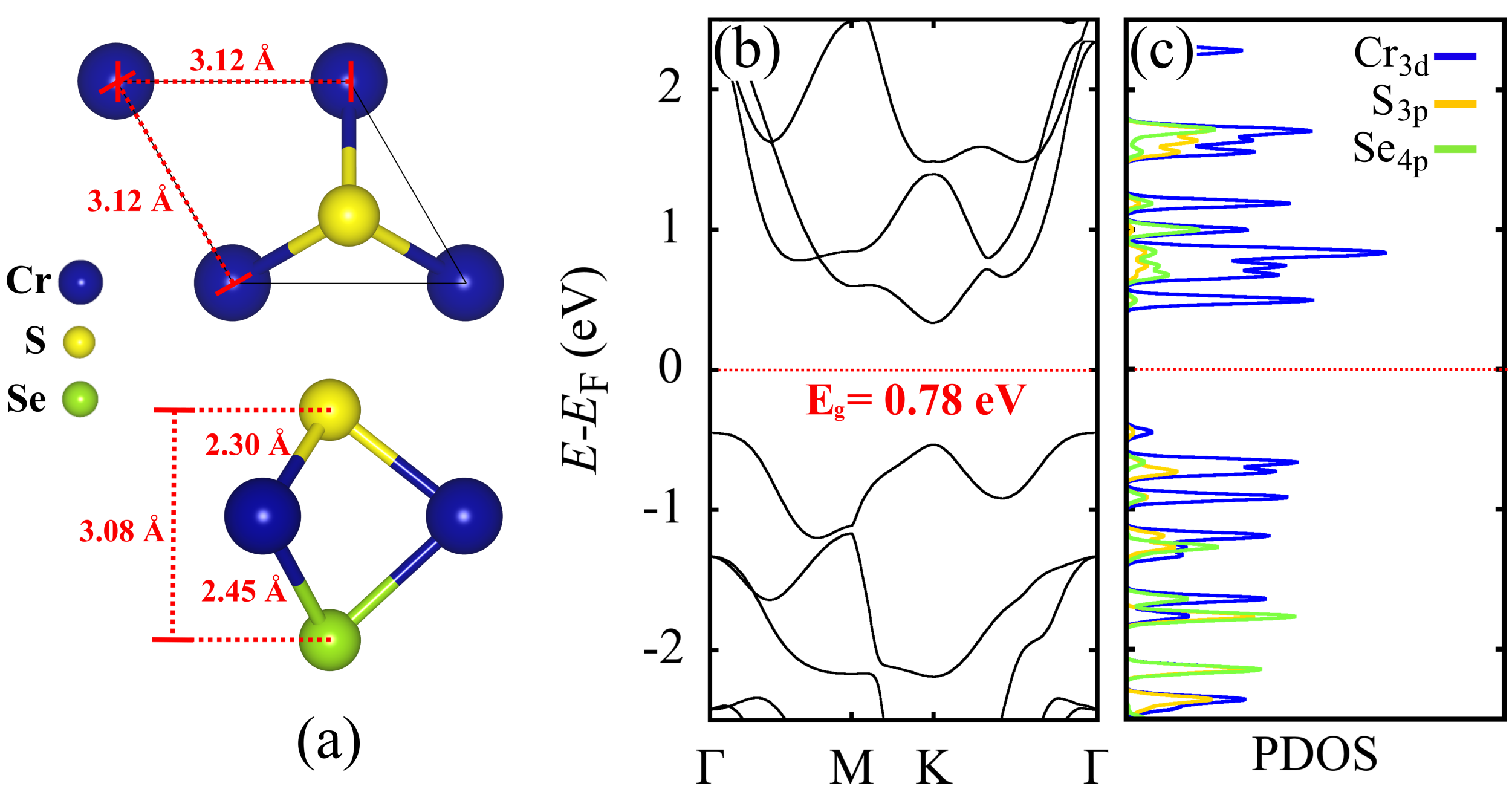

Fig. S1. Structural and electronic properties of pristine 2D Janus CrSSe. (a) Relaxed unit cell showing representative interatomic distances (Cr–Cr, Cr–S, and Cr–Se) and the out-of-plane thickness of the monolayer, as indicated. (b) Electronic band structure along the high-symmetry path Γ–M–K–Γ, with the Fermi level set to zero energy (red dashed line) and the corresponding band gap E_g highlighted. (c) Projected density of states (PDOS) for Cr-3d, S-3p, and Se-4p orbitals. Blue, yellow, and green spheres denote Cr, S, and Se atoms, respectively.

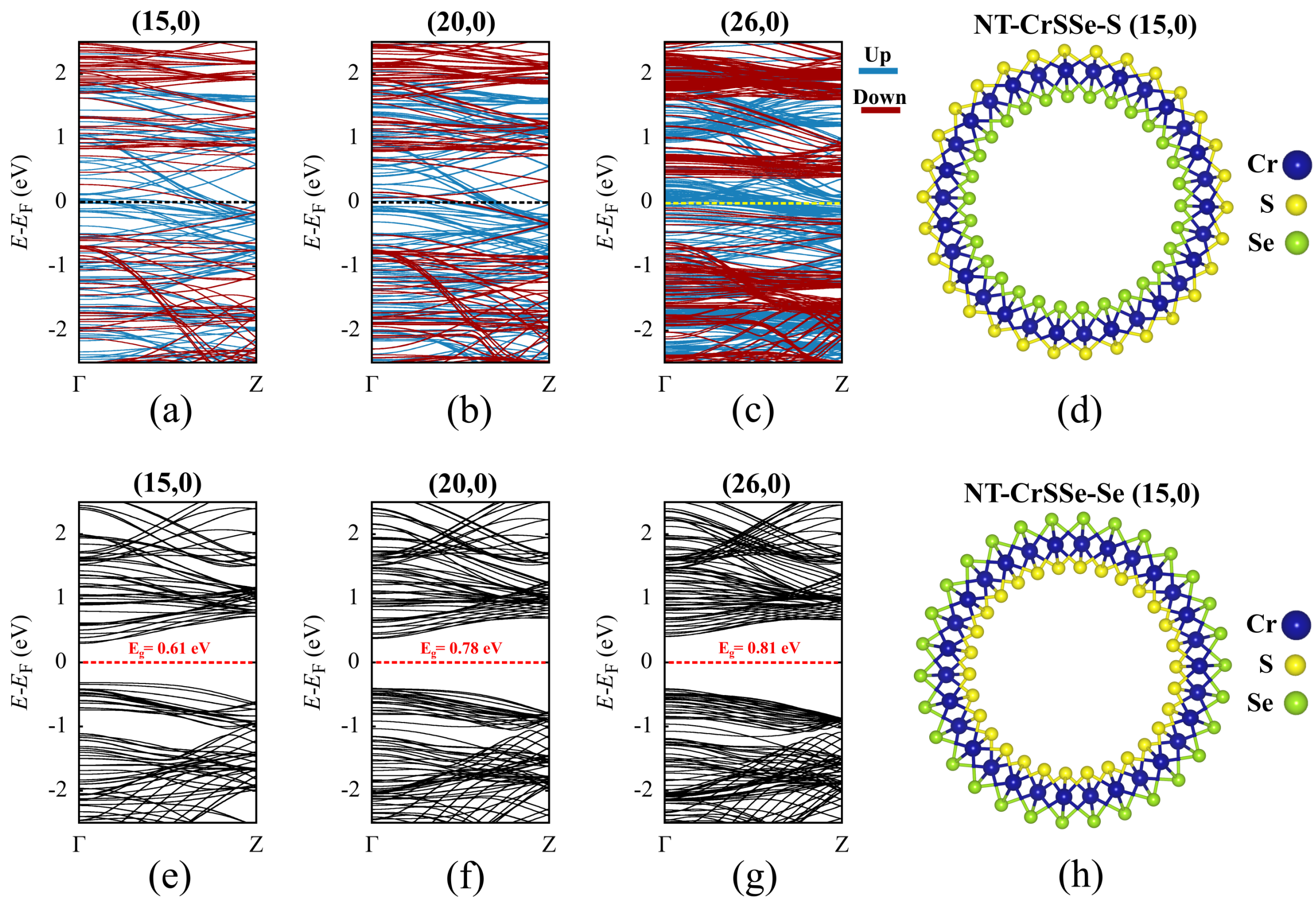

Fig. S2. Electronic band structures of Janus CrSSe zigzag nanotubes with opposite terminations. (a–c) Spin-polarized band structures of CrSSe-NT-S$(n,0)$ for $n = 15$, 20, and 26, with spin-up and spin-down channels shown in blue and red, respectively. (e–g) Band structures of CrSSe-NT-Se$(n,0)$ for the same chiralities, with the corresponding band gaps E_g indicated. The Fermi level is set to zero and the dispersion is shown along Γ–Z. (d,h) Cross-sectional views of the optimized CrSSe-NT-S$(15,0)$ and CrSSe-NT-Se$(15,0)$ nanotubes, respectively. Blue, yellow, and green spheres denote Cr, S, and Se atoms.

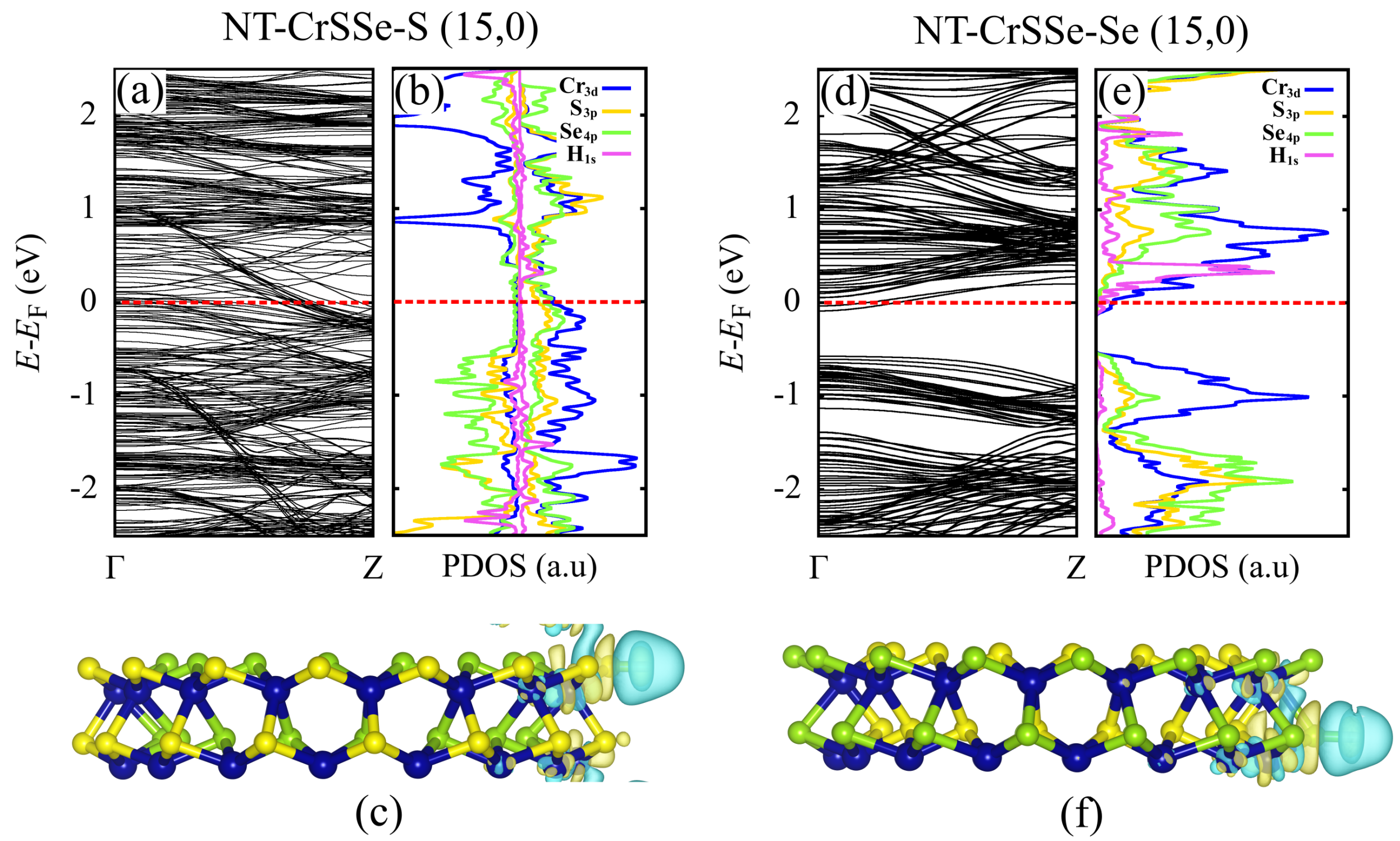

Fig. S3. Electronic structure and charge redistribution upon H* adsorption on Janus CrSSe nanotubes. (a,b) Band structure along the 1D Brillouin-zone path Γ–Z and the corresponding projected density of states (PDOS) for the CrSSe-NT-S(15,0) nanotube with adsorbed H*. (d,e) Same quantities for the CrSSe-NT-Se(15,0) nanotube with adsorbed H*. The Fermi level is set to zero energy and indicated by the red dashed line; PDOS contributions from Cr-3d, S-3p, Se-4p, and H-1s states are shown. (c,f) Optimized adsorption geometries together with the charge-density-difference isosurfaces, highlighting charge accumulation (cyan) and depletion (yellow) induced by H* adsorption on the two opposite terminations.

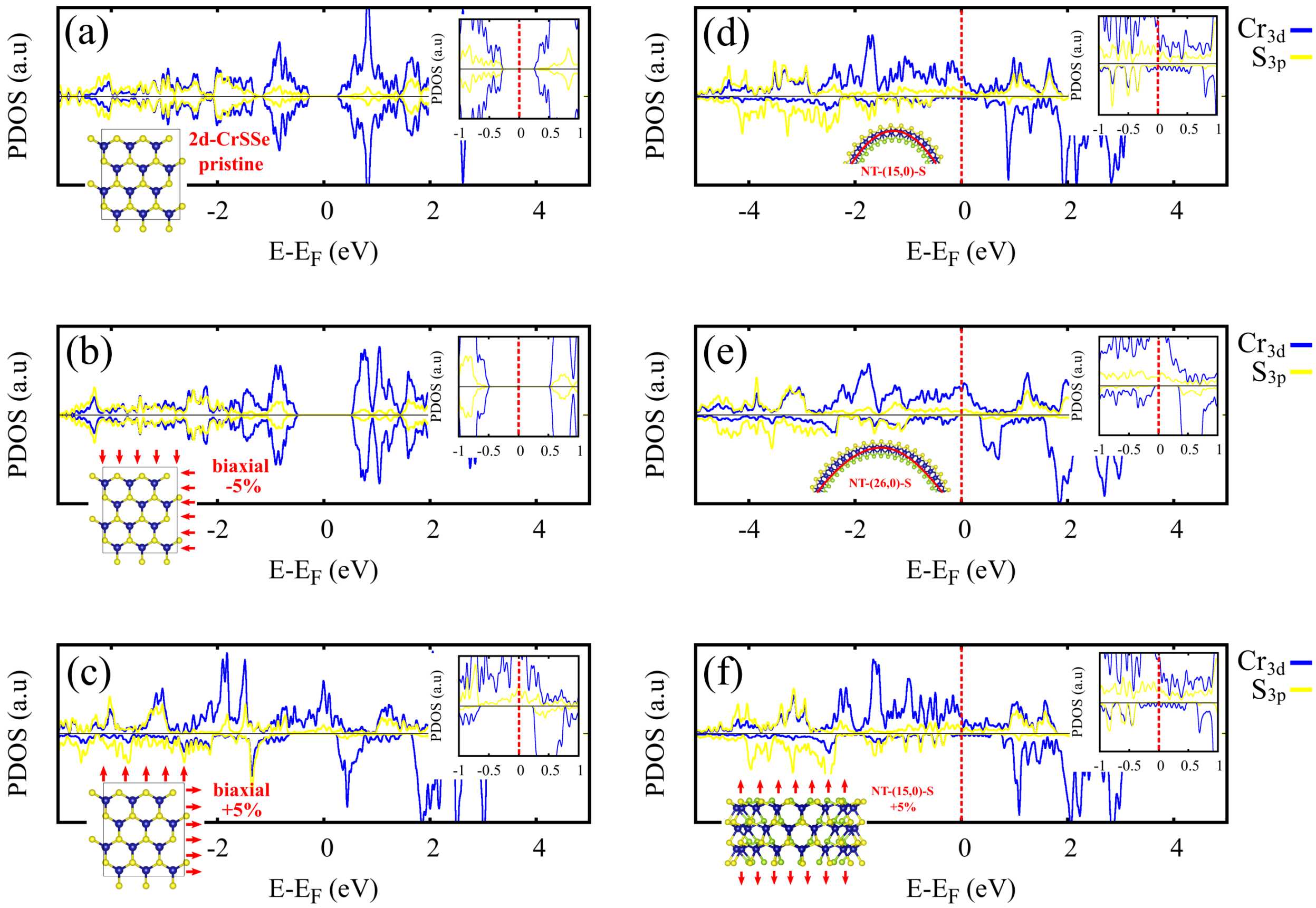

Fig. S4. Projected density of states (PDOS) of the S-3p and Cr-3d orbitals for 2D Janus CrSSe and S-terminated CrSSe nanotubes under different structural conditions. (a–c) Pristine 2D CrSSe and the corresponding structures under -5% biaxial compressive and $+5\%$ biaxial tensile strain, respectively. (d,e) Pristine CrSSe-NT-S$(15, 0)$ and CrSSe-NT-S$(26, 0)$ nanotubes, respectively, and (f) CrSSe-NT-S$(15, 0)$ under $+5\%$ axial tensile strain. The vertical red dashed line marks the Fermi level, E_F. The upper-right insets magnify the PDOS within -1 to $+1$ eV around E_F, while the structural schematics illustrate the corresponding strain or curvature conditions. Variations in the S-3p/Cr-3d states near E_F highlight the modification of orbital hybridization induced by strain and curvature.

Geometrical coordinates of representative models

The optimized Cartesian coordinates of representative key structures are provided as separate in VASP POSCAR/CONTCAR format and can be downloaded from the reference [I]. The included structures are: pristine 2D CrSSe orthorhombic 3×2 supercell, CrSSe-NT-S(15,0), and CrSSe-NT-Se(15,0). The nanotube models correspond to the relaxed S- and Se-terminated zigzag configurations discussed in the manuscript. All coordinates are given

in Å.

[I] P. Alves da Silva Autreto and J. R. Gomez Quispe, “Files for Supporting Information of Modulating the Electronic Structure and Basal-Plane Adsorption of Janus CrSSe through Mechanochemical Activation”. Zenodo, May 20, 2026. doi: 10.5281/zenodo.20316344.
